# High-resolution UV spectroscopy of the chiral molecule 1-phenylethanol

**DOI:** 10.1039/d5cp02854j

**Published:** 2025-09-26

**Authors:** Shilpa Yadav, JuHyeon Lee, Gerard Meijer, Sandra Eibenberger-Arias

**Affiliations:** a Fritz-Haber-Institut der Max-Planck-Gesellschaft Faradayweg 4-6 14195 Berlin Germany eibenberger@fhi-berlin.mpg.de; b Fachbereich Physik, Freie Universität Berlin Arnimalle 14 14195 Berlin Germany

## Abstract

The rotationally resolved excitation spectrum of the S_1_ ← S_0_ electronic transition of the chiral molecule 1-phenylethanol is measured *via* laser-induced fluorescence detection in a cold, seeded molecular beam. The rotational constants and structure of the S_1_ state are determined by fitting 419 spectral lines. The transition dipole moment is found to have predominant projections along the *b* and *a* inertial axes with only a small contribution along the *c*-axis, in agreement with *ab initio* calculations. Using two-color (1 + 1′) resonance-enhanced multiphoton ionization the S_1_ excited state lifetime is determined as 70 ± 18 ns.

## Abstract 2

High-resolution UV spectroscopy of the S_1_ ← S_0_ transition of 1-phenylethanol reveals the rotational energy level structure of the S_1_ state.

## Graphical abstract

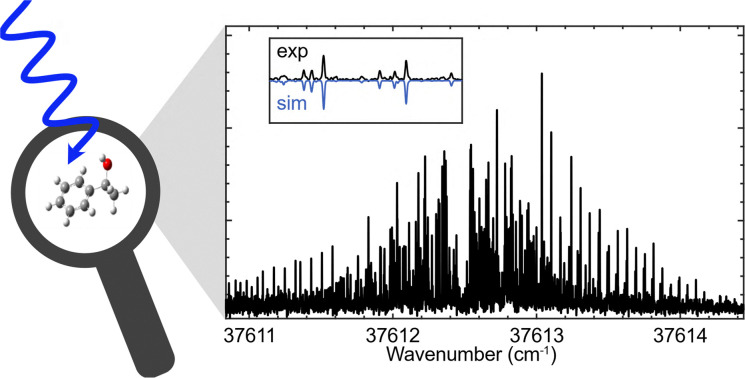

## 1. Introduction

Chiral molecules interact differently with chiral environments, leading to enantiomer-specific outcomes in biological and chemical systems. These effects are central to processes such as drug binding, asymmetric catalysis, and molecular recognition,^1,2^ which makes studying these stereospecific interactions highly relevant. Gas-phase spectroscopy enables such studies by allowing chiral molecules to be examined in isolation, free from solvent effects, while also permitting the controlled introduction of specific binding partners. This makes it a powerful tool for chiral analysis and for probing non-covalent interactions under well-defined conditions.^3,4^

Recent advances in chiral research have been driven by novel spectroscopic methods that rely solely on strong electric dipole interactions, such as photoelectron circular dichroism^5^ and microwave three-wave mixing (M3WM).^6,7^ An intriguing extension of M3WM is enantiomer-specific state transfer (ESST), that enables enantiomer-selective population control in rotational states using tailored microwave fields.^8,9^ Recent work has demonstrated near-complete quantum state control of the chiral molecule 1-indanol in a triad of rotational states using a combined UV-microwave scheme.^10,11^ This approach is applicable to all chiral molecules with *C*_1_ symmetry that have spectroscopically well-characterized ground and excited states.

A relatively light, structurally rigid chiral molecule that appears to be a promising candidate for ESST studies is 1-phenylethanol. This molecule is of practical significance due to its widespread use in the fragrance industry for its odor profile, and in pharmaceutical synthesis as a chiral intermediate.^12–14^ It is liquid at room temperature, it has a UV chromophore, it has *C*_1_ symmetry and its ground state rotational energy level structure has been well characterized.^15^ However, the rotational structure in its first electronically excited singlet state, the S_1_ state, has not been explored yet.

Structurally, 1-phenylethanol consists of a phenyl ring bonded to a chiral center with a hydroxyl and a methyl group. This structure permits internal rotation about both the C–C and O–H bonds. Theoretical calculations predict multiple low-energy conformers,^16^ yet experiments consistently reveal only a single conformer, named *t*_I_,^15,17^ even when using helium as a carrier gas. As interaction with the carrier gas can catalyze conformational conversion,^18^ the barrier that the higher energy conformers have to overcome to relax to the *t*_I_ structure is likely lower than the binding energy of the 1-phenylethanol–He van der Waals complex. The *t*_I_ conformer and its 1 : 1 and 1 : 2 complexes with water have been characterized using IR–UV double-resonance spectroscopy, to investigate hydrogen bonding and microsolvation effects.^17^ Ground state microwave spectroscopy has provided accurate rotational constants for both the monomer and its hydrated clusters.^15^ The vibronic structure of the S_1_ ← S_0_ transition has been studied by UV and ECD spectroscopy, revealing Herzberg–Teller intensity borrowing and Duschinsky mixing.^19^

In this study we present the rotationally resolved origin band of the electronic S_1_ ← S_0_ transition of 1-phenylethanol. This UV spectrum is obtained *via* laser-induced fluorescence (LIF) spectroscopy in a cold molecular beam. The rotational constants of the S_1_ state are obtained by fitting the measured spectral lines and are compared to the outcome of *ab initio* calculations. The geometric structure of the S_1_ state and the orientation of the transition dipole moment in the molecular frame are determined. Using two-color (1 + 1′) resonance-enhanced multiphoton ionization (REMPI) the radiative lifetime of the S_1_ state has been measured.

## 2. Experimental

**Fig. 1:**
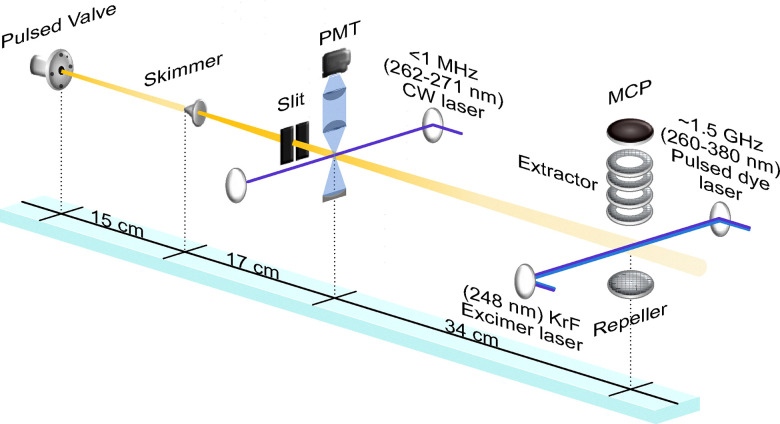
Scheme of the experimental setup. Molecules are seeded in helium and introduced into the vacuum chamber *via* a pulsed valve. A cold molecular beam forms downstream of the skimmer and is further collimated by a 2 mm slit. Vibrationally resolved spectra are recorded using resonance-enhanced multiphoton ionization (REMPI) coupled with time-of-flight mass spectrometry, with ions detected by a microchannel plate (MCP) detector. Rotationally resolved spectra are obtained *via* laser-induced fluorescence (LIF), using a continuous-wave (CW) laser for excitation and a photomultiplier tube (PMT) for fluorescence detection.

A sketch of the experimental setup is depicted in Fig. 1. A heated racemic sample of 1-phenylethanol (∼47 °C) is entrained in helium carrier gas at a backing pressure of 3 bar. This mixture is expanded into vacuum through a pulsed valve (General Valve, Series 9) with a 0.9 mm nozzle diameter. After passing through a 2 mm diameter skimmer, a supersonically cooled molecular beam is formed, which is then collimated and enters the detection chambers. The pulsed valve operates at repetition rates of 10 Hz for the REMPI and 30 Hz for the LIF measurements.

Vibrationally resolved UV spectra are recorded using one-color (1 + 1)-REMPI time-of-flight mass spectrometry. Both excitation and ionization are achieved using the frequency-doubled output of a dye laser (Sirah PSCAN-D-18, ∼0.05 cm^−1^ resolution, 260–380 nm, few mJ pulse energy), pumped by a pulsed Nd:YAG laser (355 nm, 250 mJ). Ions generated in the interaction region are guided through a time-of-flight tube and detected with a microchannel plate (MCP). The wavelength of the pulsed dye laser is continuously monitored using a wavemeter (HighFinesse WS6-600). Additionally, the lifetime of the S_1_ excited state is measured using a two-color (1 + 1′)-REMPI scheme.^20^ For this, the dye laser excites the S_1_ ← S_0_ transition while a KrF excimer laser (248 nm) ionizes the excited molecules. The mass-selected ion signal is recorded as a function of the time delay between the two lasers. Neutral density filters are used to attenuate the laser power to ensure any background signal from either laser alone is negligible.

Rotationally resolved UV spectra are acquired using LIF detection. A continuous-wave, frequency-quadrupled diode laser (TOPTICA, TA-FHG pro) is employed for S_1_ ← S_0_ excitation, operating in the 262–271 nm range with a narrow linewidth of <1 MHz. The wavelength is precisely measured using a HighFinesse WS8-10 wavemeter, which provides an absolute accuracy of approximately 20 MHz in the UV region. The LIF signal is collected using a photomultiplier tube (PMT, Hamamatsu R7154), which is positioned perpendicular to both the laser propagation axis and the molecular beam. To suppress scattered laser light, a high-reflectivity mirror coated for 266 nm (>∼275 nm transmission) is placed in front of the PMT.

To limit the transverse velocity spread of the molecular beam, a 2 mm slit is placed 2.5 cm upstream from the LIF detection point, thereby reducing Doppler broadening. To prevent Doppler shifts in the spectrum, it is essential to align the laser beam perpendicular to the molecular beam axis. To ensure this, a retro-reflection setup is implemented using a zero-degree mirror, allowing the laser beam to be reflected back through the interaction region. UV spectra are recorded both with and without the retro-reflected beam. When the laser is properly aligned perpendicular to the molecular beam, the resulting spectra from both configurations show identical peak positions, confirming the absence of Doppler shifts.

A conformational potential energy surface (PES) is calculated by scanning the dihedral angle corresponding to the rotation of the hydroxyl group and the phenyl ring. Geometry optimizations of the energetically most stable conformer (*t*_I_) are carried out at various levels of theory. The resulting ground state rotational constants are compared with experimental values to identify the level of theory that yields the best agreement. This procedure leads to the selection of the dispersion-corrected B3LYP-D3BJ functional^21,22^ with the 6-311++G(d,p) basis set, which accurately accounts for the π–electron interaction between the OH group and the phenyl ring. Geometry optimization of the *t*_I_ conformer in the ground state (S_0_) is performed at this level. The excited state (S_1_) geometry is optimized using time-dependent density functional theory (TD-DFT), and both vertical and adiabatic excitation energies are calculated. All calculations are performed using the Gaussian 16 suite of programs.^23^

The rotationally resolved spectrum is fitted using PGOPHER.^24^ The process begins by generating an initial spectrum simulation using the experimentally known S_0_ rotational constants and the *ab initio* calculated S_1_ rotational constants. Tentative assignments of experimental transitions are made by comparing the experimental spectrum with the simulated one. These assigned transitions are then used to refine the rotational constants in the S_1_ state. The updated constants yield an improved simulation, which in turn facilitates the assignment of further transitions. This cycle of assigning transitions and refining constants is repeated until all measured transitions are assigned and accurately reproduced by the simulation.

## 3. Results and discussion

**Fig. 2:**
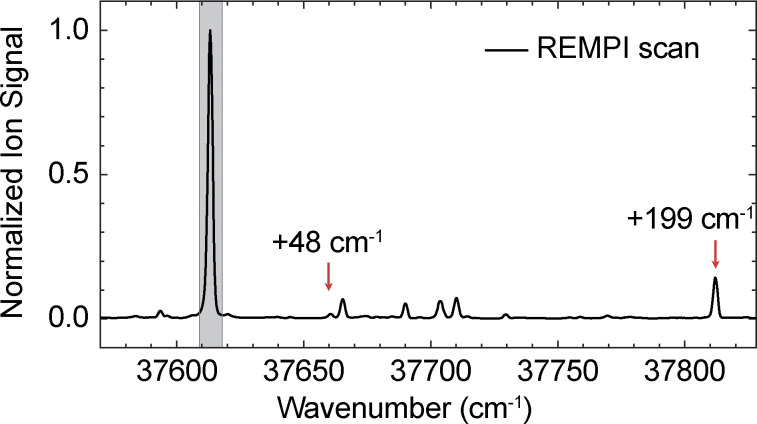
Vibrationally resolved (1 + 1)-REMPI spectrum of the S_1_ ← S_0_ transition of 1-phenylethanol recorded with a step size of 0.7 cm^−1^.

Fig. 2 shows the vibrationally resolved UV spectrum, with the most prominent peak corresponding to the origin band of the S_1_ ← S_0_ electronic transition at 37 612 cm^−1^. Just as in previous studies, we observe only one conformer in the spectrum.^25,26^ Weak features at −19, +52, +77, and +91 cm^−1^ relative to the origin arise from dissociated water-clustered species. These features also appear on the parent mass and have also been reported upon in earlier cluster studies.^17^ The weak band at +48 cm^−1^ is assigned to the fundamental Φ–R torsional mode, and the feature at +199 cm^−1^ corresponds to the CH_3_ torsion. These assignments are supported by anharmonic computational predictions.

**Fig. 3:**
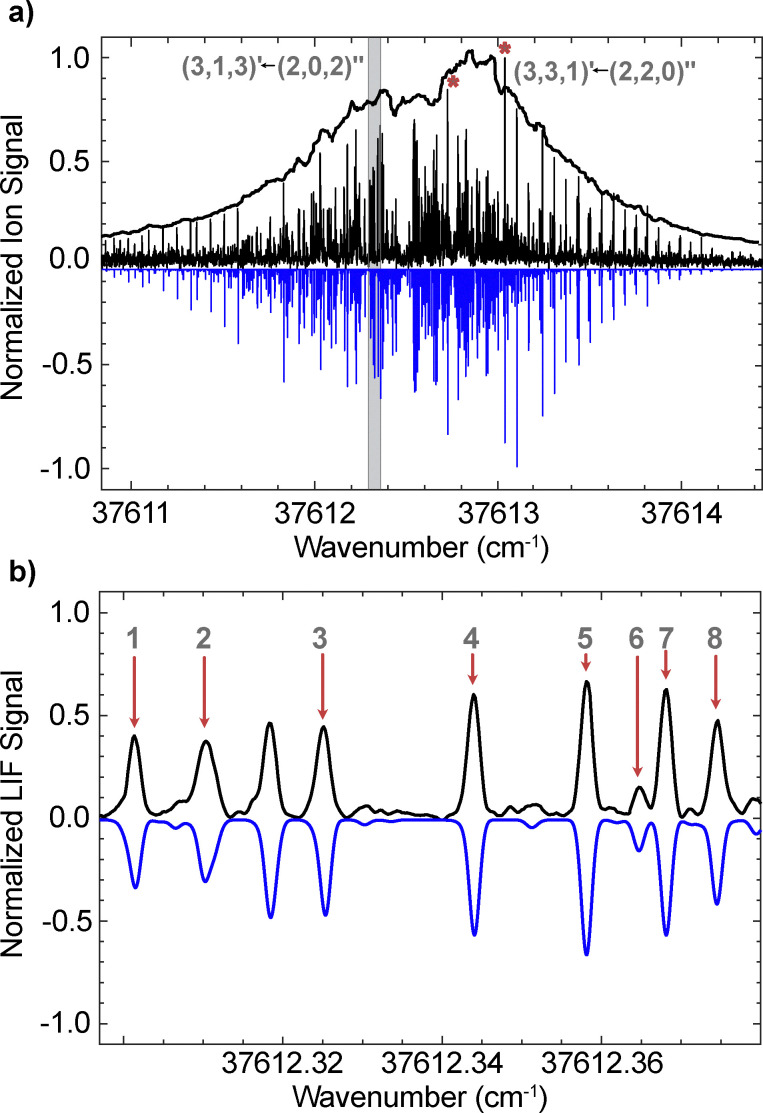
High-resolution LIF spectrum of the S_1_ ← S_0_ origin band of 1-phenylethanol. (a) The rotational band contour obtained from REMPI measurements is shown as a black trace. Superimposed are the rotationally resolved experimental transitions (black lines, pointing up) recorded *via* high-resolution LIF, along with the PGOPHER simulation (blue lines, pointing down). (b) Expanded view of the grey-shaded region in (a), highlighting the agreement between the experimental spectrum and the simulation. Peaks 1–8 correspond to the transitions: 1 – (6,0,6)′ ← (6,1,5)′′, 2 – (0,0,0)′ ← (1,1,1)′′, 3 – (5,0,5)′ ← (5,1,4)′′, 4 – (4,0,4)′ ← (4,1,3)′′, 5 – (3,0,3)′ ← (3,1,2)′′, 6 – (5,5,1)′ ← (5,5,0)′′, 7 – (2,0,2)′ ← (2,1,1)′′, and 8 – (1,0,1)′ ← (1,1,0)′′.

Fig. 3 presents the high-resolution LIF spectrum of the origin band of 1-phenylethanol. The rotational band contour observed in the (1 + 1)-REMPI measurements is shown for reference, together with the fully resolved LIF spectrum (black lines, pointing up) and the PGOPHER simulation (blue lines, pointing down). A total of 419 transitions are fitted, yielding a standard deviation of the fit of ∼5.2 MHz. From this fit, the electronic origin (*T*_00_) is determined to be at 37 612.4584(7) cm^−1^, and the rotational temperature is found to be ∼1.8 K. Also marked in the figure are two of the most intense transitions in the spectrum, both belonging to the R-branch, namely (3,1,3)′ ← (2,0,2)′′ and (3,3,1)′ ← (2,2,0)′′, using the conventional (*J*, *K*_a_, *K*_c_)′ ← (*J*, *K*_a_, *K*_c_)′′ notation. An expanded view of the shaded region is shown in the lower panel, highlighting the agreement between experiment and simulation.

The experimental spectrum is dominated by b-type and a-type transitions, with only minor contributions from c-type transitions. A detailed analysis of the relative line intensities in different parts of the spectrum indicates that the transition dipole moment (*μ*) is predominantly along the *b*-axis (*μ*_*b*_ ∼ 0.86–0.90 μ), with the *a*-axis component being about half as strong (*μ*_*a*_ ∼ 0.40–0.51 μ) and the *c*-axis component nearly an order of magnitude weaker (*μ*_*c*_ ∼ 0.06–0.14 μ). The molecular constants derived from the fit are listed in Table 1, along with theoretical predictions. The calculated absolute value and sign of the three components of the transition dipole moment are given in Debye in Table 1 and are in good agreement with the experimental observations. The differences between the ground and excited state rotational constants are Δ*A* = −109.165(14) MHz, Δ*B* = −9.546(12) MHz, and Δ*C* = −22.3722(88) MHz. These negative shifts indicate that the molecule expands along all three principal axes upon electronic excitation, with the largest change occurring along the *a*-axis. This structural expansion is attributed primarily to a slight lengthening of C–C bonds in the aromatic ring associated with the ππ* transition. The calculated rotational constants at the B3LYP-D3BJ/6-311++G(d,p) level agree with the experimental values to within 0.6% or better, validating the geometry changes inferred from the measurements.

**Table 1:** Molecular constants for 1-phenylethanol in the ground (S_0_) and excited state (S_1_). Experimental rotational constants (*A*, *B*, *C*), inertial defect (*Δ*), asymmetry parameter (*κ*), S_1_ ← S_0_ origin (*T*_00_), and standard deviation of the fit (*σ*) are given. Calculated molecular parameters at the B3LYP-D3BJ/6-311++G(d,p) level of theory are included for comparison. Experimentally, we can only determine the relative magnitude of the components *μ*_*a*_, *μ*_*b*_ and *μ*_*c*_ of the overall transition dipole moment *μ*. The sign and the absolute value of these components as obtained from the calculations are given in Debye

	Experimental^a^^b^	Calculation
Ground state (S_0_)		
*A* (MHz)	3465.5588	3471.98
*B* (MHz)	1103.96187	1099.67
*C* (MHz)	981.27316	982.48
*D*_*J*_ (KHz)	0.1072	—
*D*_*JK*_ (KHz)	0.235	—
*d*_1_ (KHz)	−0.01093	—
*d*_2_ (KHz)	0.0087	—
*Δ* (amu Å^2^)	−88.59	−90.74
*κ*	−0.901	−0.905

Excited state (S_1_)		
*T*_00_ (cm^−1^)	37 612.4584(7)	40 139.42
Δ*A* (MHz)	−109.165(14)	−104.9
Δ*B* (MHz)	−9.546(12)	−1.7
Δ*C* (MHz)	−22.3722(88)	−17.97
*Δ* (amu Å^2^)	−85.31	−86.4
*κ*′	−0.886	−0.889

Transition dipole moments		
*μ*_*a*_	0.40–0.51 μ	0.05 D
*μ*_*b*_	0.86–0.90 μ	−0.08 D
*μ*_*c*_	0.06–0.14 μ	−0.007 D

Standard deviation of fit		
*σ* (MHz)	5.2	—

a The ground state rotational and centrifugal distortion constants are taken from microwave experiments^15^ and kept fixed; the same centrifugal distortion constants are used for the S_1_ state.

b The 1*σ* standard deviations in parentheses refer to the uncertainty in the last digit.

The inertial defect shifts from ∼−88.6 amu Å^2^ in the ground state to ∼−85.3 amu Å^2^ in the excited state, indicating increased planarity upon excitation (as a planar molecule would have a zero inertial defect). This increased planarity is explained by the decrease in key dihedral angles, including a change in the OH torsion angle from ∼54° to ∼50° and an even more pronounced change in the orientation of the CH_3_ group with respect to the plane of the phenyl ring by ∼25°. These structural changes are consistent with the transition dipole moment orientation, which is found to project predominantly along the *b*-axis, with a secondary contribution along *a*- and negligible projection along *c*-axes.

**Fig. 4:**
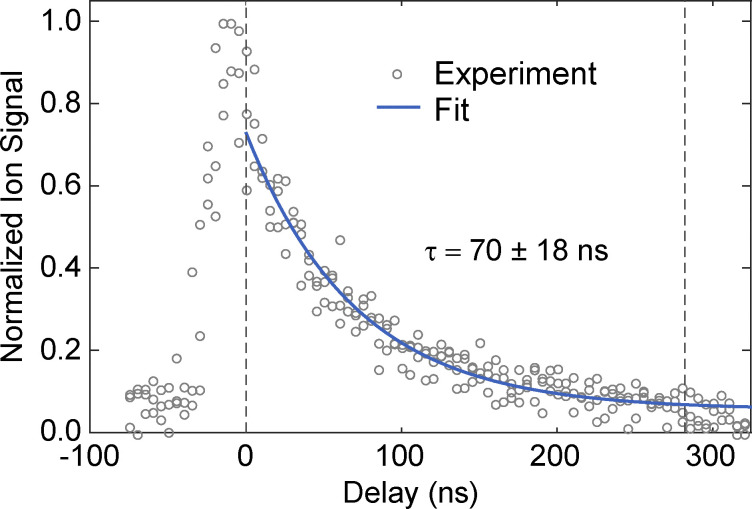
Lifetime measurement of the S_1_ excited state of 1-phenylethanol. The normalized ion signal is shown as a function of the delay between the excitation and ionization lasers in a two-color (1 + 1′)-REMPI experiment. Experimental data (gray circles) are fitted with a single exponentially decaying function (blue line), yielding a lifetime of *τ* = 70 ± 18 ns.

In our high-resolution spectra, the observed spectral linewidth of 54 MHz (full width half maximum; FWHM) is primarily determined by Doppler broadening. The next largest contribution comes from the natural linewidth of the transitions, which can be determined by measuring the radiative lifetime of the S_1_ excited state. Fig. 4 shows the ion signal as a function of the delay between timing of the dye laser, positioned on the origin band of the S_1_ ← S_0_ transition, and timing of the KrF excimer laser (248 nm). By fitting this data to a single exponentially decaying function we determine a lifetime of 70 ± 18 ns (95% confidence limit), which corresponds to a natural linewidth of only ∼2.2 MHz (FWHM). The ionizing laser provides sufficient photon energy to also ionize from a triplet state, which would result in a biexponentially decaying curve. The observed single-exponential behavior and the clear return of the ion signal to the baseline seems to indicate that there is no significant contribution from such a long-lived triplet state.

## 4. Conclusions and outlook

In this study we have characterized the electronically excited S_1_ state of 1-phenylethanol *via* high-resolution spectroscopy and *ab initio* calculations. The extracted rotational constants indicate that electronic excitation leads to a modest expansion of the molecular structure, primarily in the phenyl ring, along with increased planarity due to side-chain reorientation. The transition dipole moment is found to be oriented predominantly along the *b* and *a* axes, consistent with this structural change. With the energy level structure of 1-phenylethanol in both the S_0_ and S_1_ state known, the groundwork for future ESST experiments in our molecular beam setup[11] has been laid. When UV depletion is applied prior to ESST, R-branch lines that are fairly isolated and among the strongest can be used. These same lines can also be used for sensitive LIF detection of rotational level population after ESST. The excited state lifetime of approximately 70 ns enables that transitions with a spectral resolution down to the natural linewidth of 2.2 MHz can be recorded. Together, these findings establish 1-phenylethanol as a viable chiral target for future ESST and related quantum control applications.

## Conflicts of interest

There are no conflicts to declare.

## Data Availability

All data supporting the conclusions of this work are included in the manuscript and the raw data are available in the Edmond database (https://doi.org/10.17617/3.X2KODE).^27^
